# Molecular identification of *Brucella* species and biovars associated with animal and human infection in Iran

**DOI:** 10.30466/vrf.2018.89680.2171

**Published:** 2019-12-15

**Authors:** Maryam Dadar, Saeed Alamian, Ali Mohammad Behrozikhah, Freshteh Yazdani, Armin Kalantari, Afshar Etemadi, Adrian M. Whatmore

**Affiliations:** 1 *Department of Brucellosis, Razi Vaccine and Serum Research Institute (RVSRI); Agricultural Research, Education and Extension Organization (AREEO), Karaj, Iran; *; 2 *Animal and Plant Health Agency (APHA), Addlestone, Surrey, United Kingdom.*

**Keywords:** Brucellosis, Bruce ladder, PCR, Brucella species and biovars

## Abstract

Brucellosis is a costly contagious disease of human, domestic and wild animals. It is a serious health problem in Iran causing significant economic losses therefore, control approaches to prevent its spread are of great importance. In Iran, the species and biovars of virulent *Brucella* species are still under-reported due to the inadequate diagnostic protocols and insufficient laboratory facilities. The objective of this study was to characterize *Brucella* isolates obtained from passive animal and human surveillance in Iran from 2011 to 2018 in order to understand the current epidemiological situation of the disease. A total of 419 samples (milk, blood, cerebrospinal fluid, abomasum content and aborted fetus tissues) were collected from 65 cases/case series (human and animals) and examined bacteriologically. The initially identified *Brucella* isolates were further characterized using phenotypic and molecular approaches. All recovered isolates were either *B. abortus* or *B. melitensis*. The infection in sheep appeared to be exclusively associated with *B. melitensis,* but both *B. abortus* and *B. melitensis* were common in bovine samples. Samples from one sheep and one goat were confirmed to be infected by the *B. melitensis* vaccine strain Rev1. In spite of *B. abortus* burden in animals (14 cases in cattle and camel), brucellosis in human was predominantly associated with *B. melitensis *(15 cases). The results confirmed that *B. melitensis* biovar 1 and *B. abortus* biovar 3 remain the most prevalent biovars in Iran. This report builds a picture of the significance of different *Brucella *species in different hosts in Iran and provides applicable information for the healthcare professionals about the public health risks of brucellosis and relevant preventive strategies.

## Introduction

Brucellosis is known as a highly contagious disease of ruminants and humans caused by Gram-negative, non-motile coccobacilli of the genus *Brucella*.^1^ Among *Brucella *spp., *B. abortus*, *B. suis*, and *B. melitensis* are the most pathogenic and invasive species for human and livestock.^2^ *Brucella canis* can also cause human infection but is considered a less significant zoonotic threat.^3^ Brucellosis is still an uncontrolled public health issue in some endemic regions in the Mediterranean, northern and eastern Africa, the Middle East and parts of Latin America and Asia. More than 500,000 new human cases of brucellosis are reported annually by World Health Organization (WHO).^4^ The efficient preventive approaches have been established in several developed countries such as Australia, Northern European nations, Canada, Japan, and New Zealand,^3^ which led to brucellosis eradication. However, brucellosis still represents a threat for human and livestock worldwide,^5^ and is also categorized by the Centers for Disease Control and Prevention (CDC) as a class B bioterrorist pathogen that has historically been developed as a bio-weapon.^6^

Iran represents an endemic region for brucellosis.^7^ The geographical distribution of brucellosis in this area is constantly changing with new foci of infection emerging or re-emerging.^8^^,^^9^ Roushan *et al*. evaluated the clinical manifestations and epidemiological features of 469 adult patients suffering from brucellosis in the northern part of Iran and reported that the consumption of unpasteurized dairy products, working at a laboratory, practicing in a veterinary profession, and direct contact with livestock as the main routes for brucellosis infection.^10^ Other studies reported that the consumption of unsafe dairy products and animal husbandry as the main risk factors of brucellosis in different parts of Iran. ^8^^.^^11^ The prevalence of human and animal brucellosis have been reported in various parts of Iran such as east,^11^^,^^12^ central,^13^^,^^14^ west^15^^,^^16^ and south.^17^^,^^18^ For example, the prevalence of brucellosis from 2002-2006 was 340 per 10,000 in small ruminants and 56.00 per 10,000 in cattle. In addition, the incidence of human brucellosis was 37.00 per 100,000 in the east of Iran.^19^ In another study, it was revealed that the mean incidence rate of human brucellosis was 60.00 per 100,000 during 2001-2010 in the central part of Iran. The highest reported incidence rate in humans was 111.5 per 100,000 in 2004, while the lowest incidence rate was 40.50 per 100,000 in 2006.^13 ^In the west of Iran, the incidence of brucellosis in human reached 59.31 per 100,000 in 2013.^16^ These represent among the highest incidences for human brucellosis reported globally.^20^

A few studies have reported the isolation and characterization of *Brucella* from human or livestock samples by approaches such as classical biotyping and modern molecular approaches such as IS*711*-based (AMOS) PCR.^8^^,^^11^^,^^12^ AMOS PCR is described as a recent advance for accurate and rapid diagnosis of brucellosis that has been reported to overcome the limitations of conventional methodology. ^9^^,^^10^

A previous study by Zowghi *et al*. reported that *B. abortus* biovars 1, 2, 3, 4, 5 and 9 (predominantly 3) were isolated from sheep and a small number of cattle. Also, *B. melitensis* biovars 1, 2 and 3 (predominantly 1) were isolated from sheep, goats, cattle, camels, dogs, and humans.^21^ However other *Brucella* species such as *B. neotomae*, *B. suis*, *B. ovis* and *B. canis* were not detected.^21^ In another study it was revealed that *Brucella* isolates causing abortion in small ruminants were predominantly *B. melitensis* biovar 1, with a smaller number of *B. melitensis *biovar 2 isolates and a single isolate of *B. abortus* biovar 3.^22^ The present study aims to further evaluate and update the presence and the nature of *Brucella* spp. in Iran. 

## Materials and Methods 

**Sample collection. **A number of 419 samples including 151 milk samples (126 cows, one camel, 16 sheep and eigth goats), 58 human blood samples, three human cerebrospinal fluid (CSF) samples, 46 abomasal contents (32 cows, 14 sheep), 94 bovine lymph nodes and 67 tissue samples (kidney, liver, abomasum, spleen, heart and lung) from the aborted fetuses (26 sheep, 39 cows, two goats) were collected from 2011 to 2018. Samples were submitted to the Department of Brucellosis of the Razi Vaccine and Serum Research Institute (Karaj, Iran) from 2011 to 2018. Cows, sheep, and goats with a history of abortion were examined in the farms. Samples from all visceral organs (liver, abomasum content, lungs, kidneys, spleen, and heart) were collected in sterile plastic bags and preserved at – 20.00 ˚C for *Brucella* culture and isolation. Human cases of brucellosis were patients referred with clinical complaints to the RVSRI Department of Brucellosis with symptoms compatible with brucellosis, and positive Wright and 2ME tests. Milk and serum samples were stored at – 20.00 ˚C until analysis. The animals and human samples were collected from different location of Iran such as Mashhad, Sari, Kashan, Qom, Kerman, Ilam, Shahrekord, Tehran, Shiraz, Karaj, Gharchak, Yazd, Marvdasht, Zanjan, Rey, Semnan and Garmsar from 2011 to 2018.

***Brucella*** **isolation. **For the isolation and identification of *brucella,* bacteriological assays were performed under appropriate protection in safety hoods at the RVSRI Department of Brucellosis. All individual milk samples, abomasum content and aborted fetal organs, as well as blood samples, were subjected to bacterial culture. Primary isolation of *Brucella *spp*.* was performed by inoculating the samples on a *Brucella* selective supplement containing polymyxin B (2,500 IU), bacitracin (12,500 IU), nystatin (50,000 IU), cycloheximide (50.00 mg), nalidixic acid (2.50 mg) and vancomycin (10.00 mg) all from Oxoid (Basingstoke, UK) and inactivated 5.00% horse serum in *Brucella* agar (Himedia, Mumbai, India). The inoculated media were incubated for 10 days at 37.00 ˚C with 10.00% CO_2_. Milk samples were centrifuged for 15 min at 3,500 rpm and afterward, sediments and the creamy upper layer were cultured. After 14 days of incubation, the bacterial cultures were discarded if no growth was visible. Typical colonies of *Brucella *spp*.* were sub-cultured and subjected to further analysis to obtain full identification and biotype.

**Biotyping. **Classical biotyping was performed according to the procedure described by Alton *et al*.^23^ *Brucella* monospecific antisera A and M and *Brucella* reference phage of Tbilisi (Tb) were routinely prepared and used for diagnosis and analysis in the Razi Vaccine and Serum Research Institute. A panel of biotyping tests such as CO_2_ dependence, H_2_S production, agglutination with specific *Brucella* antisera, growth in media containing thionin and basic fuchsin, agglutination by acriflavine, and lysis by specific phages were performed^24^ and the results were interpreted according to the documented data.^23^

**Molecular typing. **Genomic DNA was extracted by heat-treating a loopful of bacterial colony dissolved in 300 µL of molecular-grade water at 100 ˚C for 15 min.^25^ The suspension was vortexed and centrifuged at 13,000 *g* for 5 min and then the supernatant containing DNA was collected and stored at − 20.00 ˚C until later use.^26^ The DNA concentration was evaluated by measuring the DNA absorbance at 260 nm. After that, the extracted DNA was subjected to IS*711*-based (AMOS) PCR for *Brucella *spp.^27^ Species-level molecular identification was also performed using seven primer pairs (Table 1) in a multiplex PCR (Bruce-ladder) to reveal different *Brucella *spp. under the following conditions: initial denaturation at 95.00 ˚C for 5 min, 30 cycles of 95.00 ˚C for 30 sec, 56.00 ˚C for 90 sec, 72.00 ˚C for 3 min and a final extension step at 72.00 ˚C for 10 min.^28^ The amplified products were resolved by electrophoresis using a 1.50% agarose gel.

## Results

*Brucella* isolates (n=161) were recovered from 48 out of 65 examined cases/case series. These included isolates from human blood (47/58), human CSF (1/3), ovine aborted fetuses (9/26), ovine milk (1/16), bovine milk (49/126), bovine aborted fetuses (3/39), bovine lymph nodes (44/94), bovine abomasum contents (5/32), goat milk (1/8) and camel milk (1/1). Isolated bacteria exhibited common phenotypic features typical of *Brucella* spp. All isolates grew in 10.00% carbon dioxide (CO_2_) after 5 to 14 days incubation at 37.00 ˚C. Bacteria isolated were Gram-negative and formed small honey colored, translucent and shiny colonies with a smooth surface. Isolates were characterized to the biovar level and identity confirmed to the species/vaccine level for all isolates by the use of AMOS PCR and Bruce-ladder. Isolates represented either *B. abortus*, *B. melitensis* or *B. melitensis* vaccine strain Rev1. 

**Fig. 1:**
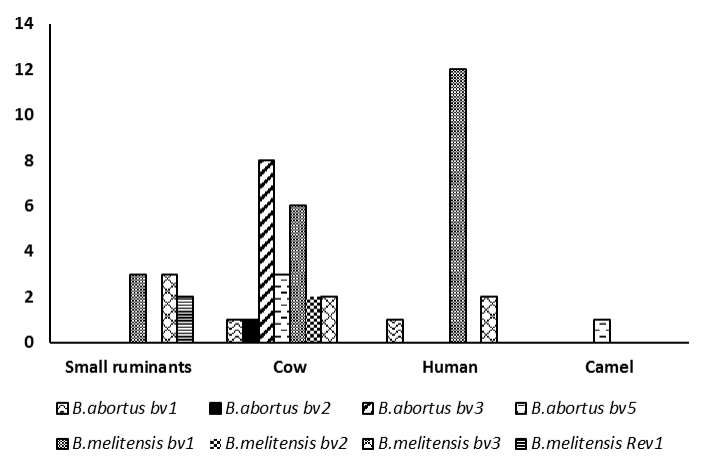
The frequency of *B. abortus* and *B. melitensis* biovars in different examined samples

***Brucella abortus. ***A number of 89 *Brucella* isolates identified as *B. abortus *which they were isolated from 15 cases (cattle 13 cases, human and camel each one case), (Fig. 1). Biotyping was consistent with the presence of *B. abortus* biovars 1 (two cases), 2 (one case), 3 (eight cases) and 5 (four cases). As expected, only the biovar 1 and 2 isolates gave the 498 bp *B. abortus *specific band in AMOS PCR, which detects only biovars 1, 2 and 4.^29^ However all isolates were confirmed as *B. abortus* in the Bruce-ladder PCR with PCR products of 1682, 794, 587, 450 and 152 bp in size (Table 1). 

**Table 1:** The list of primer pair names and sequences and the expected amplicon sizes for different *Brucella* species

**Strains**	**Primer **	**Primer sequence (5-3’)**	**Target gene**	**Amplicon size (bp)**	**Ref.**
***B. abortus***	*IS711* *AB*	TGCCGATCACTTTCAAGGGCCTTCAT GACGAACGGAATTTTTCCAATCCC	*IS711*	498	
***B. melitensis***	*IS711* *BM*	TGCCGATCACTTTCAAGGGCCTTCATAAATCGCGTCCTTGCTGGTCTGA	*IS711*	731	
***B. abortus*** ***B. melitensis*** ***B. *** ***melitensis *** **Rev.1**	BMEI0998f BMEI0997r	ATCCTATTGCCCCGATAAGG GCTTCGCATTTTCACTGTAGC	Glycosyltransferase, gene *wboA*	1682	
***B. abortus*** ***B. melitensis*** ***B. *** ***melitensis *** **Rev.1**	BMEI0535f BMEI0536r	GCG CATTCTTCGGTTATGAA CGCAGGCGAAAACAGCTATAA	Immunodominant antigen, gene *bp26*	450	
***B. abortus*** ***B. melitensis*** ***B. *** ***melitensis *** **Rev.1**	BMEI1436f BMEI1435r	ACGCAGACGACCTTCGGTAT TTTATCCATCGCCCTGTCAC	Polysaccharide deacetylase	794	
***B. abortus*** ***B. melitensis*** ***B. *** ***melitensis *** **Rev.1**	BMEII0428f BMEII0428r	GCCGCTATTATGTGGACTGG AATGACTTCACGGTCGTTCG	Erythritol catabolism, gene *eryC *	587	
***B. abortus*** ***B. melitensis*** ***B. *** ***melitensis *** **Rev.1**	BMEII0987f BMEII0987r	CGCAGACAGTGACCATCAAA GTATTCAGCCCCCGTTACCT	Transcriptional regulator, CRP family	152	
***B. melitensis***	BMEII0843f BMEII0844r	TTTACACAGGCAATCCAGCA GCGTCCAGTTGTTGTTGATG	Outer membrane protein, gene *omp31*	1071	
***B. *** ***melitensis *** **Rev.1**	BMEI0752f BMEI0752r	CAGGCAAACCCTCAGAAGC GATGTGGTAACGCACACCAA	Ribosomal protein S12, gene *rpsL*	218	

***Brucella melitensis. ***Seventy-two isolates were identified as *B. melitensis *which they were isolated from 33 cases (Fig. 1) including sheep/goats (eight cases), cows (10 cases) and humans (15 cases). *Brucella* *melitensis *isolates belonged to three biovars of those biovar 1 (22 cases) was more common than the other two biovars 2 (two cases) and 3 (seven cases). The last two *B*. *melitensis* isolated from a sheep fetus and goat milk sample were *B. melitensis* Rev 1 vaccine strain on the basis of Bruce-ladder typing. All the other isolates were identified as wild type *B. melitensis* by both AMOS-PCR with a PCR product of 731 bp and Bruce-ladder with PCR products of 1682, 794, 587, 450, 152 and 1,071 bp in size. *Brucella* *melitensis* Rev.1 vaccine strain was differentiated from the other *B. melitensis* strains through a specific additional band of 218-bp (Table 1).

**Fig. 2:**
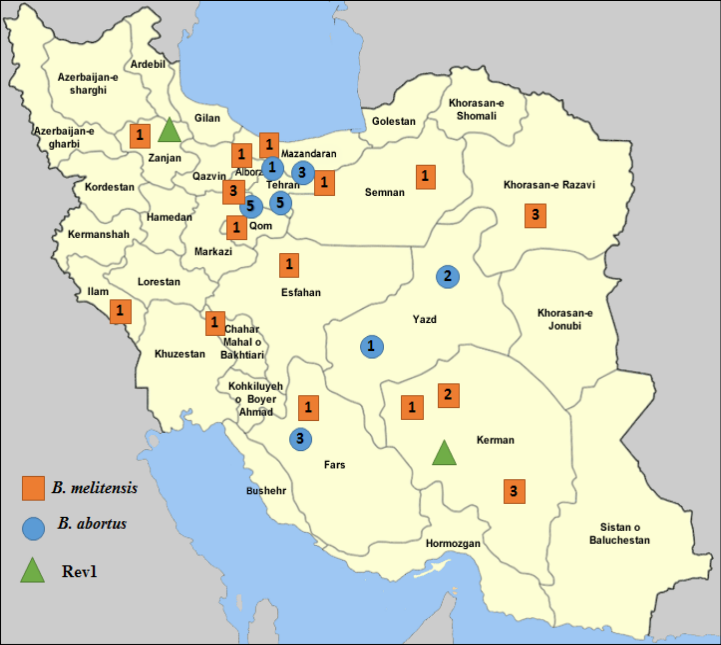
The geographical distribution of *Brucella* species/biovars of animals and humans in Iran. The numbers inside the boxes indicate the frequencies of *Brucella* biovars

**Geographical distribution of** ***Brucella*** **species/ biovars. **A map of the distribution of *Brucella* species/biovars across Iran revealed that *B. melitensis* biovar 1 is the most prevalent biovar identified in 11 provinces (Tehran, Mazandaran, Alborz, Qom, Zanjan, Esfahan, Ilam, Chahar Mahal-va-Bakhtiari, Semnan, Kerman, and Fars). *Brucella* *melitensis* biovar 2 was only identified in Kerman province, while *B. melitensis* biovar 3 was identified from samples collected from three provinces of Alborz, Kerman and Khorasan-e Razavi. *Brucella abortus* biovar 1 was detected from samples originating from two provinces of Tehran and Yazd. *Brucella* *abortus* biovar 2 was only detected in Yazd Province, biovar 3 in Tehran and Fars, while biovar 5 was identified in provinces of Tehran and Qom. In addition, Rev1 vaccine strain was isolated from samples collected from two provinces of Zanjan and Kerman (Fig. 2). 

## Discussion

Due to the endemic nature of brucellosis in some regions of Iran, the knowledge of epidemiological, and clinical features of common and virulent *Brucella *species is crucial for better diagnosis, prevention, and control of the disease. The gold standard procedure for the diagnosis of human and animal brucellosis is still the bacterial culture and the isolation of the causative agent followed by bacteriological tests and biotyping. In many Iranian studies, *Brucella* infection has been investigated using serology and PCR tests, but there are very few studies that examine the actual presence of *Brucella *species and biovars recently. For this purpose, we performed bacteriological and molecular methods (such as Bruce-ladder not previously applied in Iran) to further characterize *Brucella* biodiversity in the animal and human infected communities in Iran. The results of the present study extended our knowledge of *Brucella *species and biovars that are currently associated with this zoonosis disease in Iran. These reports also revealed the passive surveillance for brucellosis over a seven-year period showing the significant burden of both *B. abortus* and *B. melitensis *in the past few years. In terms of livestock, while *B. abortus* appeared to be largely restricted to cattle, *B. melitensis* appeared to be common in both cattle and small ruminants. This is consistent with the increasing observations of the isolation of *B. melitensis* from cattle, particularly in Africa and the Middle East^2^^,^^30^^-^^32^ and with previous observations in Iran.^21^ Human brucellosis though appears predominantly associated with *B.* *melitensis* (94.00% of cases), with a much lower burden of *B. abortus* (6.00%), consistent with the view that *B.* *melitensis* is the most significant human pathogen among *Brucella* species globally.^6^^,^^33^

According to our results, *B. melitensis* biovar 1 was the significant *Brucella* species predominantly isolated from humans with rarer isolation of *B. melitensis* biovars 3 and *B. abortus* biovars 1. These results are in accordance with the findings of the previous study from different parts of Iran, reporting *B. melitensis* biovars 1 as endemic and widely spread in humans.^21^ This type of *brucella* was also reported in more recent studies as the most prevalent isolate in humans elsewhere.^6^^,^^34^

*Brucella abortus* biovar 3 was the most prevalent type in cattle. This finding is in agreement with a previous epidemiological study performed in Iran, recognizing this biovar as the main and the most virulent in cattle.^21^ *B. abortus* biovar 3 has also been reported as a common cause of abortion in dairy cows in Europe, China, and Turkey as well as in Kenya^21^^,^^35^ though molecular studies have shown that *B. abortus* biovar 3 corresponds to multiple separate lineages.^36^

Based on our results, *B. melitensis* biovars 1, *B. melitensis* biovars 3 and *B. melitensis* Rev1 are the only species that have been isolated in the aborted sheep fetus. *B. melitensis* biovar 1 was first reported from a sheep in the center of Iran (Isfahan) and then spread in different Iranian regions, infecting sheep and goats as well as cattle, camel, dogs, and humans.^21^ The study of Ashrafganjooyi *et al*. on 700 milk samples reported *B. melitensis* biovar 1 as the most common biovar in sheep and goat milk samples.^37^ *B. melitensis* biovar 1 was also reported in Iran,^21^^,^^22^ China,^38^ Libya,^39^ Israel,^40^ Kenya^41^ and Oman,^42^ while *B. abortus* biovar 1 is present in Iran,^21^ Pakistan,^43^ and Kuwait.^44^

This study showed that *B. melitensis* biovar 3 is also common in Iran that is inconsistent with a previous report from Turkey.^45^ In the Middle East countries, *B. melitensis* biovars 3 has also been reported as the most common cause of *Brucella* infections in human.^42^ According to our data,* B. melitensis* biovar 2 was only reported in cows from Kerman. In spite of being the most prevalent biovar in China,^38^ this biovar seems to be present in a lower extent in the Middle East and Mediterranean countries. *Brucella melitensis biovar 2* was previously reported from Saudi Arabia, Iran and Turkey, ^22^^,^^42^ and our results reported the incidence of this biovar in cattle. 

*Brucella abortus* biovar 5 was isolated from the single Iranian camel sample examined in the present study. The infection of camels with *B. melitensis* biovar 3 and *B. abortus* biovar 6 has been reported in Western Sudan.^46^ Another study reported the presence of *B. abortus* biovar 1 from camel milk in Kuwait.^44^ Generally, camel brucellosis has been reported in different parts of Iran, Saudi Arabia, Oman, Kuwait, Sudan, Iraq, Egypt, Somalia and Libya as well as in the United Arab Emirates.^47^ Although, both *B. abortus* and *B. melitensis *can infect camels,^46^^-^^48^ Zaki reported *B. abortus* as the most common cause of brucellosis in camels.^49^ Further work is needed to determine the brucellosis burden in Iranian camels. 

Two isolates of *B. melitensis* Rev1 from an ovine foetus and caprine milk confirmed the propensity of Rev1 to be shed in milk and causing abortion in the small ruminants particularly if the timing of vaccination is not optimal.^50^ The attenuated *B. melitensis* Rev 1 strain is currently administrated as the exclusive vaccine for the prevention of brucellosis in sheep and goat in Iran. The full- and reduced-doses of Rev 1 have been suggested as safe and effective approaches for controlling small ruminant brucellosis. Taken together, our results, utilizing both classical methods and newly introduced molecular approaches, demonstrated the frequency of brucellosis infection in Iran and reflects the spread of various *B. melitensis *and *B. abortus *biovars. In spite of this update, there are significant gaps in the different Iranian literature on the epidemiology of *Brucella* in cattle, sheep, camels, and human, therefore, further works are required to fully understand the epidemiology of this disease. This knowledge will ultimately support the design of potential brucellosis control programs and preventive strategies in Iran.
